# What Happens to the Immune System after Vaccination or Recovery from COVID-19?

**DOI:** 10.3390/life11111152

**Published:** 2021-10-29

**Authors:** Bruna T. Tiyo, Gabriela J. H. Schmitz, Marina M. Ortega, Laís T. da Silva, Alexandre de Almeida, Telma M. Oshiro, Alberto J. da S. Duarte

**Affiliations:** Laboratório de Investigação Médica em Dermatologia e Imunodeficiências (LIM 56), Hospital das Clínicas HCFMUSP, Faculdade de Medicina, Universidade de São Paulo, BR. Av. Dr. Enéas Carvalho de Aguiar, 470. IMT II, 3° Andar, São Paulo 05403-000, SP, Brazil; brunatiyo@usp.br (B.T.T.); marina.ortega@usp.br (M.M.O.); lais.teodoro@usp.br (L.T.d.S.); alisalmeida@gmail.com (A.d.A.); telma.oshiro@hc.fm.usp.br (T.M.O.); adjsduar@usp.br (A.J.d.S.D.)

**Keywords:** COVID-19, cellular response, humoral response, vaccines, recovered patients, transmission, correlates of protection, herd immunity

## Abstract

Due to its leading role in fighting infections, the human immune system has been the focus of many studies in the context of Coronavirus disease 2019 (COVID-19). In a worldwide effort, the scientific community has transitioned from reporting about the effects of the novel coronavirus on the human body in the early days of the pandemic to exploring the body’s many immunopathological and immunoprotecting properties that have improved disease treatment and enabled the development of vaccines. The aim of this review is to explain what happens to the immune system after recovery from COVID-19 and/or vaccination against SARS-CoV-2, the virus that causes the disease. We detail the way in which the immune system responds to a SARS-CoV-2 infection, including innate and adaptive measures. Then, we describe the role of vaccination, the main types of COVID-19 vaccines and how they protect us. Further, we explain the reason why immunity after COVID-19 infection plus a vaccination appears to induce a stronger response compared with virus exposure alone. Additionally, this review reports some correlates of protection from SARS-CoV-2 infection. In conclusion, we reinforce that vaccination is safe and important in achieving herd immunity.

## 1. Introduction

Coronavirus disease 2019 (COVID-19) forced the world scientific community to find answers to limitless questions about a disease incomprehensible until a short time ago. In recent months, the understanding of severe acute respiratory syndrome coronavirus-2 (SARS-CoV-2) immunopathogenesis has improved, making possible important advances in patient management to reduce mortality. Likewise, the rapid progress in vaccine development and mass vaccination have had a profound impact on reducing the number of infections, hospitalizations and deaths. The emergence of new variants of concern (VOCs), which have increased viral transmissibility, is posing new challenges due to the possibility of immune evasion and a reduction in the effectiveness of vaccines currently in use.

In this context, the immune system plays an important role not only in the evolution and resolution of the infection but also in protection against the virus. Despite the large amount of knowledge and data already accumulated about COVID-19, questions remain. Therefore, our intention was to review how the immune system handles contact with SARS-CoV-2 by infection or vaccination, mainly if protective immunity is induced and to what degree. Additionally, a description of different types of vaccines and their roles, along with immune correlates of protection from COVID-19 were the gaps we explored.

Here, we present a brief overview of current knowledge about the role of the immune system in response to COVID-19, as well as aspects related to immunization and its importance in stopping the pandemic.

## 2. The Immune Response after SARS-CoV-2 Contact

### 2.1. Innate Immune Response against SARS-CoV-2

Our body has several barriers to protect us from invading microorganisms, such as physical barriers (skin and mucous membranes), chemical barriers (the action of antimicrobial peptides and reactive oxygen species), and immune system responses (innate and specific adaptive immune response) [1]. Despite this powerful apparatus, SARS-CoV-2 is able to overcome these barriers and establish an infection.

The most common route of SARS-CoV-2 infection is exposure to respiratory fluids [2]; accordingly, studies have suggested that approximately 100 airborne particles may be sufficient to initiate a SARS-CoV-2 infection [3]. Upon inhalation, SARS-CoV-2 employs cell-surface angiotensin-converting enzyme-2 (ACE2) as a receptor for entry into host cells [4]. ACE2 is more highly expressed in the oropharyngeal mucosal and endothelial cells, oral and alveolar tissues, kidneys and the gastrointestinal tract [5,6].

SARS-CoV-2 replication in host cells elicits a series of events focused on viral clearance. Innate immune system cells, such as dendritic cells, monocytes, macrophages, neutrophils, eosinophils, mast cells and natural killers, can recognize evolutionarily conserved features of pathogens, termed pathogen-associated molecular patterns (PAMPs) [7] and damage-associated molecular patterns (DAMPs). These patterns are host-derived molecules released from damaged or dying cells [8]. From these interactions, soluble mediators (e.g., cytokines, chemokines, effector mechanisms, and the complement system) can be produced to inhibit the establishment of an infection [9]. Although knowledge in this area is still limited, some studies have shown higher plasma levels of DAMPs in COVID-19 patients than in healthy controls, which may be evidence of DAMPS’ participation in acute respiratory distress syndrome (ARDS) [10,11]. This initial inflammation can eliminate the infected cells before the virus spreads, resolving the infection or, alternatively, the inflammatory mediators produced by host innate immune defences may cause dysregulated responses, leading to damage to lung and vascular tissues, for example [12,13].

Several innate immune cells present sensors known as Toll-like receptors (TLRs). Some studies have suggested the potential beneficial effects of TLR agonist administration, at least during the early phases of COVID-19 infection, to enhance the innate and adaptive immunity and help the viral clearance [14,15]. However, the interaction of SARS-CoV-2 proteins with TLR2 [16,17], TLR4 [18,19,20] and TLR6 [21,22] has been observed dysregulating type I interferon [23] and inflammatory responses, leading to hyperactive cytokine release, termed cytokine storm [24], a condition observed in most severe COVID-19 patients.

Cytokine storms are marked by the activation of myeloid cells that secrete inflammatory cytokines and chemokines (TNF-α, IFN-γ [25], IL-6, IL-8 [26,27,28], IL-1α [29], IL-1 receptor antagonist (IL-1Ra), IP-10, MCP-1, MCP-3 [30] and CCL3 [31]). These inflammatory molecules have been associated with T cell depletion and disease severity, since excessive and persistent inflammation can cause lung injury with the development of ARDS and, in extreme cases, multiple organ failure that may lead to death [23,32,33]. Furthermore, the induction of tissue damage is exacerbated by the impaired type I IFN response in infected cells, which allows viral replication [32,34].

All of these observations have prompted researchers to develop strategies to modulate proinflammatory cytokine levels, and a possible treatment to reduce the incidence of ARDS in COVID-19 patients has been studied [16,35,36,37].

The transition between innate and adaptive immune responses is crucial to COVID-19 outcomes [38]. In this regard, aspects of the SARS-CoV-2-specific adaptive immune response on disease severity and/or patient recovery will be discussed in the next section.

### 2.2. Adaptive Immune Response against SARS-CoV-2

If SARS-CoV-2 overcomes the first line of defence, the most refined and complex immune response [39], the adaptive immune response mediated by B and T cells via antigen-presenting cells (APCs) is crucial to the body’s defence.

Naïve T cells are activated and proliferate after they recognize SARS-CoV-2 peptides presented through major histocompatibility complex (MHC) molecules on the surface of infected cells, such as respiratory epithelial cells or APCs [40,41]. Activated CD8+ T cells may present a cytotoxicity profile, releasing perforin and granzymes to induce apoptosis of infected target cells [42]. In turn, CD4+ T cells can produce cytokines that influence the activation and differentiation of other immune cells, including APCs, helping them destroy ingested virus, CD8+ T lymphocytes kill infected target cells, and B cells secrete SARS-CoV-2-specific antibodies, including immunoglobulin (Ig)M and IgG [42].

In most cases, both humoral and cellular adaptive immune responses lead to virus clearance and the establishment of immunological memory, resulting in patient recovery [42]. Alternatively, the host immune response can be dysfunctional and suboptimal, leading to worse clinical patient conditions [43]. Different clinical outcomes have been studied, but they may depend on several characteristics of the host, including the presence of comorbidities (diabetes, cardiovascular disease, hypertension); variations in genes associated with SARS-CoV-2 replication and immunomodulatory processes (differential expression of ACE2 receptor and genes associated to inflammation); and HLA polymorphisms associated with severe cases of infection (HLA-B*46:01 allele) [44,45].

Thrombocytopenia and lymphopenia are observed in some individuals infected with SARS-CoV-2, especially those who were admitted to intensive care units (ICUs) [46,47,48,49]. Mechanisms have been proposed to explain the reduction of platelet counts, such as hematopoietic cells’ viral invasion that could lead to inhibition of hematopoiesis, decreasing platelet production. Besides, the presence of immune complexes and autoantibodies could lead to platelet destruction and lung injury, which could promote platelet activation and aggregation, generating microthrombi, increasing platelets consumption [50]. Regarding lymphopenia induction, T cells may exhibit functional exhaustion [46] or activated profile in the presence of cytotoxic CD8+ T cells, collaborating with tissue damage [38,51,52].

SARS-CoV-2-specific T cell responses have been observed in the peripheral blood of COVID-19 patients with ARDS two weeks after symptom onset. In addition, SARS-CoV-2-specific CD4+ T cells were found to produce Th1 cytokines and exhibit a central memory phenotype, while CD8+ T cells express a cytotoxic profile [53]. Additionally, although the authors were not able to relate antigen-specific T cell responses to disease severity, they found evidence for a negative correlation between viral loads and CD4+ T cell counts [53]. In this respect, Mathew et al. (2020) reported that in hospitalized COVID-19 patients, three different patterns of adaptive immune response with mortality occurred in all of them. In the first immunotype associated with disease severity, they observed activated CD4+ T cells, dysregulation of CD8+ T cells, and the presence of plasmablasts; the second immunotype was not correlated with disease severity and revealed less CD4+ T cell activation, the presence of effector-like CD8+ T cells and memory B cells; finally, the third immune response pattern showed a negative correlation with disease severity and was defined by lack of activated T and B cell responses [54]. These examples indicate the complex interplay between the adaptive immune response and SARS-CoV-2 infection.

Regarding individuals who had recovered from COVID-19, studies have detected the presence of SARS-CoV-2-specific memory T cell responses 1 to 8 months after infection in the peripheral blood of individuals who had varying COVID-19 severity levels [52,55,56]. In addition, the authors observed that T cell responses of COVID-19 recovered donors are directed against the membrane, spike and nucleocapsid SARS-CoV-2 proteins, with the production of TNF-α and IFN-γ by CD4+ T cells and CD8+ T lymphocytes displaying a cytotoxic profile by the expression of granzyme B and perforin [55].

The humoral adaptive immune response is another important part of protective immunity against infections. Most individuals seroconvert within 7 to 14 days of SARS-CoV-2 infection [57,58,59], when IgM levels rise rapidly; then become undetectable generally within 2 months after diagnosis [60]. Thus, virus-specific IgG levels start to be detected approximately 10 days after the onset of symptoms, reaching their peak in 21–25 days [61].

Typically, IgG and neutralizing antibody levels start declining within 2–3 months of the patient’s recovery, although individuals with detectable IgG titres 8 months after symptom onset have been reported [56]. Although antibodies gradually decay over time after infection, SARS-CoV-2-specific memory B cells, which are able to expand and differentiate rapidly into antibody-secreting plasmablasts [62], have been identified in recovered COVID-19 patients 1 to 7 months after the onset of symptoms [56,63,64], and the frequency of B cells was higher than that in healthy controls [63].

Some of COVID-19 infected individuals develop the so-called "long COVID". These individuals experienced long-term symptoms, including cognitive impairment, fatigue, dyspnoea, flu-like, ageusia and anosmia, which can extend for months after COVID-19 onsets [65,66]. These residual symptoms could disable individuals from returning to their normal life. Although the mechanisms behind the persistence of symptoms have not been identified yet, they may be related to sequelae of organ damage, hyperactivation of the immune system/generation of autoantibodies, and the presence of comorbidities or adverse effects from medication administered [67].

In general, the immune response against SARS-CoV-2 is highly heterogeneous among individuals and circulating antibody titres are not predictive of T cell memory [56]. Studies have shown that regardless of disease severity, most recovered individuals have detectable specific adaptive humoral and cellular immune responses to SARS-CoV-2 [68,69]. Answers to the questions of whether these responses provide protection against severe COVID-19 reinfection, mainly to new SARS-CoV-2 variants, and if there is a relation between immune system responses and the occurrence of “long COVID”, remains to be explored. 

## 3. Immune Response Induced by COVID-19 Vaccines

COVID-19 has not only impacted public health but also affected society and economies worldwide. SARS-CoV-2 spread quickly, causing more than 4.5 million deaths and numerous sequelae, prompting researchers around the world to improve treatments and therapies to reduce mortality. Currently, vaccine development has become an urgent focus and to date, there are seven vaccines approved by the World Health Organization (WHO), 112 in clinical development and 185 in the preclinical phase [70]. Among the approved vaccines, different platforms have been used: inactivated virus, viral vectors, and RNA-based vaccines.

Inactivated virus vaccines use the classical approach, and scientists have produced many commercial vaccines with this platform. To generate this type of vaccine, the virus is cultivated in cells for amplification and inactivated with chemicals, UV light or heating. Thus, the entire inactivated virus provides a wide antigenic repertoire for the immune system, which hypothetically can be advantageous in the case of immune escape due to virus mutations [71,72]. CoronaVac (Sinovac Research and Development) and BBIBP-CorV (Sinopharm/China National Biotec Group Co/Beijing Institute of Biological Products) vaccines, both approved by the WHO, were based on SARS-CoV-2 inactivation.

Viral vector-based vaccines use non-replicating viruses, in general adenoviruses, modified by structural gene deletion [73], which are capable of introducing encoding viral antigens, such as the spike protein/RBD, into the host cell. In turn, the infected cell produces and releases these viral antigens, stimulating the immune response [73]. The production of this type of vaccine is simple; thus, it does not require adjuvants [74]. However, people who may have previously been exposed to other adenoviruses could have some vectors neutralized by pre-existing vectorial immunity [73]. Ad26.COV2.S and ChAdOx1 (chimpanzee adenovirus) were considered to decrease the probability of this cross-reaction. The use of adenovirus as a non-replicating viral vector is a technique applied in the approved vaccines AZD122 ChAdOx1-S (University of Oxford-AstraZeneca), Ad26.COV2.S (Janssen Pharmaceuticals by Johnson & Johnson) and Ad5-nCoV (Convidecia, CanSino Biologics Inc., Beijing Institute of Biotechnology).

RNA-based vaccines, specific for spike proteins, work by introducing the mRNA sequence into the host cells, and the sequence contains the code to produce viral proteins that will stimulate a specific immune response. This technology has the advantages of high efficiency and fast platform production, making it a favourable alternative to traditional vaccines [75]. Since mRNA is a very unstable molecule and easily degraded, especially in the presence of RNases, lipidic nanoparticles (LNPs) are used to deliver this molecule, which can protect and facilitate mRNA delivery and translation in the cytosol [70,71,74]. Due to the instability mentioned above, this vaccine requires an ultra-low temperature for storage. Examples of mRNA-based vaccines are BNT162b2 (Pfizer-BioNTech) and mRNA-1273 (Moderna), which were the first ones widely distributed.

One of the relevant questions about approved vaccines is how the immune system responds to these vaccines. Generally, all of them are capable of stimulating an immune response and are efficacious against SARS-CoV-2, even at different levels. 

Vaccines based on inactivated viruses work classically promoting the activation of APCs that, in presence of IFN-γ, stimulate CD4 T-cells, leading both to secretion of antibodies by B lymphocytes and activation of CD8 T-cells that promote the death of infected cells [74]. Viral vector-based vaccines simulate a viral infection through SARS-CoV-2 transgene expression, mimicking the characteristics of the natural infection, stimulating a strong antibody production and cytotoxic response, which leads to infected cells destruction [76]. In turn, the mRNA molecules, products of mRNA-based vaccines, are taken up by APCs and are recognized by TLR, which activates type I interferon, that potentialize the stimulation of Th1 response. Also, similar to a viral infection, the antigenic protein is produced by host cells favoring presentation via MHC I, which stimulates a cytotoxic response [77]. Thus, the type of vaccine can modulate the immune system in different ways, leading to more or less potent immune responses.

In this context, comparing previously calibrated data from Phase III trials of some currently used vaccines, Earle and colleagues demonstrated that the levels of neutralizing antibodies produced after completing the vaccination schedule are different according to the vaccine. It was observed that inactivated virus vaccines presented efficacy approximately 50%; viral vector-based vaccines showed efficacy close to 70%, and mRNA and protein subunit-based vaccines achieved efficacy above 90% [78].

Cellular response plays an equally important role in the establishment of the vaccine response. However, the comparison of the immune profile stimulated by different vaccine platforms is very difficult, since cell diversity and immune components are involved and the measurement of immune parameters can be performed by different techniques (see Table 1). Thus, it is important to emphasize the need of standardized protocols for the assessment of cellular response induced by vaccines.

In this sense, the single-cell RNA sequencing (scRNA-seq) approach is a powerful technique used to explore the heterogeneity of immune cells, allowing to identify rare cells that expand after immunization or infection, and to discovery panels of high-affinity antigen-specific antibodies [79]. Exploring this technology, Kramer and colleagues analyzed the single-cell profiling of the mRNA-based vaccine BNT162b2 (Pfizer-BioNTech) antigenic response. Data revealed a novel expanding population of non-canonical memory CD4 and CD8 T-cells following vaccination [80]. In addition, Cao and colleagues also explored this technique and described the immune dynamics during immunization with the viral vector based-vaccine Ad5-nCoV that revealed enhanced cellular immunity and boosted SARS-CoV-2 specific antibodies [81]. Data derived from the scRNA-seq technology has the potential to describe novel markers of protection following anti-SARS-CoV-2 vaccination.

Table 1 summarizes some important information about the seven main vaccines already approved by WHO.

Despite the drastic reduction in the number of infections and world relief after mass vaccination began, a relevant concern is whether approved vaccines are effective against VOCs. These new variants, Alpha (B.1.1.7), Beta (B.1.351), Gama (P.1) and Delta (B.1.617.2), were derived from genetic mutations of the original SARS-CoV-2 strain, the wild-type that surged in Wuhan, China, in December 2019. The first variant was identified in the United Kingdom, followed by South Africa, Brazil and India [82], and these variants are more infectious with a high transmission rate.

Such increase in the transmissibility of these variants is related to mutations in the S gene, whose product, the spike protein, is responsible for virus interaction with the ACE2 receptor in the host cell. Since most approved vaccines are based on the spike protein, it is of utmost importance for public health to evaluate the effectiveness of vaccines against these variants.

In general, studies published to date show that there is a reduction in vaccine protection against some of these VOCs. In an extreme case, a clinical study demonstrated that full vaccination with ChAdOx1 (University of Oxford-AstraZeneca) was not able to protect against the Beta variant in mild-moderate COVID-19 [97]. On the other hand, Ad26.COV2.S vaccine (Janssen Pharmaceuticals) and BNT162b2 (Pfizer-BioNTech) respectively showed 81.7% and 75% efficacy against this variant [98,99]. For the Alpha variant, ChAdOx1 (University of Oxford-AstraZeneca), BNT162b2 (Pfizer-BioNTech) and Ad26.COV2.S (Janssen Pharmaceuticals) vaccines showed a slight effectiveness reduction, with 70%, 90% and 80% of protection, respectively [98,99,100]. For the Gamma variant, CoronaVac (Sinovac Research and Development) and Ad26.COV2.S (Janssen Pharmaceuticals) vaccines did not show significant reduction in protection [98,101]. Finally, concerning the Delta variant, it was shown that BNT162b2 (Pfizer-BioNTech) and mRNA-1273 (Moderna) vaccines did not elicit antibody neutralization responses against this variant, which demonstrated 6.8 times more resistance to neutralization [102]. On the other hand, another group demonstrated that sera from BNT162b2 (Pfizer-BioNTech) vaccinated individuals were capable of neutralizing the Delta variant viruses at titres of at least 1:40 [103].

Despite the reduction in neutralizing capacity of variants by vaccine-induced sera, it does not seem to compromise vaccine protection. Although some cases reports demonstrate infection by the variants in fully vaccinated individuals [104], these events are rare. Therefore, once COVID-19 severe cases continue to be reduced, it seems that currently used vaccines are quite effective. New variants were expected to emerge after the COVID-19 outbreak, and while progress in developing and disseminating vaccines has been made, there are no variants completely resistant to the vaccines to date.

It is important to emphasize that all WHO-approved vaccines have presented acceptable effectiveness against SARS-CoV-2 with neutralizing antibody production and cooperation with a meaningful purpose: protecting people from COVID-19.

## 4. The Immunity and Virus Transmission after Vaccination

Since the first confirmed case of novel coronavirus in November 2019, the SARS-CoV-2 virus has spread rapidly worldwide. To bring this pandemic to an end, a large share of the world needs to be immunized against the virus, and the safest way to achieve this protection is with a vaccine. However, there is an important question left to be answered: “What is the chance of transmitting the virus after vaccination?” Therefore, in this part of this review, we tried to answer this question by discussing COVID-19 transmission from cellular and humoral responses after the first and second doses of different types of vaccines, VOCs, and advantages and disadvantages of inhaled and intramuscular COVID-19 vaccines.

According to Mallapaty (2021), several vaccines have been shown to provide high protection against COVID-19 [105]. In addition, much evidence has demonstrated that they can considerably reduce the risk of SARS-CoV-2 transmission. Concerning the protective effect, vaccinated individuals who become infected are up to 78% less likely to spread the virus to household members than unvaccinated people [106].

Further, Painter et al. (2021) reported a key observation: rapid and universal induction of SARS-CoV-2-specific CD4+ T-cells after the first mRNA vaccine dose in naive individuals [106]. This observation is extremely important given the gradual development of antigen-specific CD8+ T-cells also observed by other groups [107,108], which reach maximal levels after the second vaccine dose. These data point to the immunological benefit of two vaccine doses in SARS-CoV-naïve subjects and emphasize coordination between the different arms of the adaptive immune response following mRNA vaccination. In addition, the preferential induction of Th1, Tfh, and central memory-like T-cells, in concert with robust humoral immunity, indicates that the immune response is specifically focused on the main features of long-term antiviral immunity, which are likely to provide lasting protection against SARS-CoV-2 [109].

However, there are reports of COVID-19 transmission after the first and second doses of some types of vaccines [110,111]. Allam and Sayed (2021) reported a case in which a man (47 years old) from Saudi Arabia who received the first dose of BNT162b2 (Pfizer-BioNTech) became infected with COVID-19 and transmitted the infection to his family. This was the first case in that country of a person receiving the first dose of this vaccine who became infected afterwards. The BNT162b2 (Pfizer-BioNTech) vaccine was shown to elicit effective humoral and cellular responses a week after the second dose [112], and in the case of this patient, it seems that the virus was transmitted to him between Day 3, when he tested negative, and Day 12, when he became symptomatic. Although the first dose may have reduced the infection´s impact, it did not prevent the virus from spreading. Once his family confirmed that they had not come in contact with anyone else, this patient was, in fact, the primary source of infection for them. This case report confirms that the first dose of the mRNA vaccine BNT162b2 (Pfizer-BioNTech) is not sufficient for preventing SARS-CoV-2 infection or transmission [110].

Since the viral load of the index case is the most important risk factor for transmission [113], McEllistrem et al. (2021a) evaluated the effect of a single dose of a BNT162b2 (Pfizer-BioNTech) vaccine on viral loads among 10 individuals who developed asymptomatic COVID-19 while residing at a Veterans Affairs Pittsburgh (Pennsylvania) Community Living Center [114]. Five residents without a prior diagnosis of COVID-19 received the vaccine’s first dose, and all of them became positive by detection of SARS-CoV-2 in nasopharyngeal samples between 12-15 days after vaccination. The other five residents were unvaccinated prior to diagnosis. The comparison between vaccinated and unvaccinated individuals concerning the mean log10 viral load was significantly higher in unvaccinated residents (9.5) than in vaccinated residents (7.1). Although the five vaccinated individuals became positive after vaccination, these results showed that the single dose of a BNT162b2 (Pfizer-BioNTech) vaccine may help control outbreaks, but not stop them, since viral load is linked to transmission.

In February 2021, the first breakthrough infection occurred in a fully Comirnaty-vaccinated (BNT162b2, Pfizer-BioNTech, Mainz, Germany/New York, United States) health care worker (HCW) with high levels of neutralizing antibodies for the SARS-CoV-2 Beta (B.1.351) variant. Subsequently, the infection was transmitted to his unvaccinated spouse [115]. The HCW in his early 60s received the second dose 21 days after the first dose, according to recommendations [116], and he became positive 26 days after the booster dose, although full vaccination efficacy has been shown as early as 7 days after the second dose [117]. In this case, the HCW acquired high levels of anti-spike antibodies 33 days after the second dose and 7 days after the RT-PCR-positive swab [115], although this range of antibody levels was seen after complete vaccination with mRNA-based vaccines. Therefore, the risk of transmission by fully vaccinated individuals can occur with their close contacts [115]. Additionally, in Barnstable County (Massachusetts), which had several public events, 469 COVID-19 cases occurred among Massachusetts residents between July 6 and July 25. Overall, 346 (74%) individuals fully vaccinated [BNT162b2 (Pfizer-BioNTech) (159; 46%); mRNA-1273 (Moderna) (131; 38%); and Ad26.COV2.S (Janssen Pharmaceuticals) (56; 16%)] reported symptoms. From 133 individuals, 119 (89%) were diagnosed with the Delta variant, which is highly transmissible [118].

Regarding the CoronaVac (Sinovac Research and Development), the administration of at least one dose of this vaccine showed effectiveness among HCWs in Manaus (Brazil) against symptomatic SARS-CoV-2 infection in the setting of epidemic Gama variant transmission [119]. Additionally, Li et al. (2021), aiming to find measures to block community SARS-CoV-2 transmission, studied the CoronaVac (Sinovac Research and Development) and BBIBP-CorV (Sinopharm/China National Biotec Group Co/Beijing Institute of Biological Products) vaccines against the Delta variant, which has been associated with high transmissibility [120]. In this study were included 366 participants during the outbreak of the Delta variant in May 2021 in Guangzhou city, China. They observed that a single dose of a vaccine was not sufficiently protective (vaccine effectiveness: 13.8%) against the Delta variant. On the other hand, the vaccine effectiveness for full vaccination with two doses was estimated to be 59.0%, markedly higher than the single-dose [120]. Some of reported studies were performed before detection of the Delta variant, which has a significantly higher transmission capacity than other strains and can be more easily spread by vaccinated individuals. In fact, this can explain the rebound of the virus in many countries, including the United Kingdom (UK), where the proportion of vaccinated individuals is high and some prevention and control measures are still in place [121].

Currently, there is a lack of studies regarding the Delta variant, although Lopez-Bernal et al. (2021) found that both the BNT162b2 (Pfizer-BioNTech) and AZD122 ChAdOx1-S (University of Oxford-AstraZeneca) vaccines are slightly less effective against the Delta variant than Alpha [122]. This finding could also represent an increase in the transmissibility of the Delta variant, but these data need further exploration [105]. In Israel, where 60% of the population was fully vaccinated with BNT162b2, cases of the Delta variant have risen sharply. Therefore, a third (booster) dose of the BNT162b2 (Pfizer-BioNTech) vaccine has been approved in this country aimed at reducing transmission and curbing disease severity [123].

Additionally, vaccine administration routes have advantages and disadvantages in relation to virus transmission. Certainly, it should be noted that intramuscular COVID-19 vaccines, which are currently administered, have an important limitation: the lack of mucosal immunization, since the primary route for SARS-CoV-2 transmission is the respiratory mucosa in the respiratory tract [124]. 

According to the U.S. Centers for Disease Control and Prevention (CDC), SARS-CoV-2 is first transmitted between people via inhalation of small respiratory droplets from infected individuals and contact with infected surfaces [91]. The ingestion of droplets into the lung leads to a lower respiratory tract illness ranging from mild respiratory infection to ARDS [12]. Thus, if the location of the initial infection is primarily through the lungs, the best route of vaccine administration seems to be inhalation delivery directly to this organ.

Vaccines administered by intramuscular injection are designed to produce an IgG response, preventing viremia and COVID-19 syndrome. On the other hand, preclinical studies of adenovirus and mRNA vaccines found persistent virus in nasal swabs, suggesting that vaccinated patients, while asymptomatic, may still become infected and transmit live virus from the upper airway [125,126,127]. To effectively prevent viral replication within mucosal primary target cells, adequate local production of secretory IgA (SIgA) is necessary. Consequently, Mercado and collaborators (2020) concluded that protection in both the upper and lower respiratory tracts will be required to prevent transmission and disease in humans [126,127]. In this regard, several COVID-19 inhaled vaccine candidates are in development, and they have shown good results in preclinical studies [111]. There are six vaccines that have progressed to clinical trials: 1) AdCOVID sponsored by Altimmune [128]; 2) MV-014-212 (Meissa) [129]; 3) Coroflu (Bharat Biotech; Precision Virologics; University of Wisconsin) [130]; 4) Ad5-nCoV (CanSino Biologics) [131]; 5) AZD12222 (AstraZeneca) [132] and 6) COVI-VAC (Codagenix Inc.) [133].

According to Mallapaty (2021), vaccinated individuals who became infected were less likely to pass the infection to household members than unvaccinated individuals [105]. Several studies found that vaccination with mRNA-based vaccines in individuals who had previously been infected elicits a strong and rapid immune response [87,134]. Harris and collaborators (2021) studied 365,000 households in UK and predicted that infected individuals were 40–50% less likely to spread the infection if they had received at least one dose of BNT162b2 (Pfizer-BioNTech) or AZD122 ChAdOx1-S (University of Oxford-AstraZeneca) vaccine at least three weeks previously [135]. Salo et al. (2021) demonstrated that spouses of infected HCWs in Finland who had received one dose of BNT162b2 (Pfizer-BioNTech) or mRNA-1273 (Moderna) vaccine were 43% less likely to become infected than spouses of unvaccinated health workers [136].

These cases can be explained by humoral and cellular immune responses. Tut et al. (2021) reported the results of an investigation into the immune responses of staff and residents at long-term care facilities (LTCFs) in England after a single dose of BNT162b2 (Pfizer-BioNTech) or ChAdOx1 nCoV-19 (University of Oxford-AstraZeneca) vaccines, when previous natural infection was detected in 30 (24%) of 124 participants [137]. This study demonstrated that previous infection increased the magnitude and quality of the adaptive immune response after a single dose of vaccine. Antibody titres were 34 times higher after vaccination in previously infected residents than before vaccination. This enhanced antibody response in those with a history of infection was also observed by Krammer et al. (2021) and Manisty et al. (2021) [134,138]. In addition, Angyal et al. (2021) observed that people with a history of SARS-CoV-2 infection have 15-fold higher humoral responses after vaccination against the Beta (B.1.531) variant than participants with no previous infection [139].

Regarding cellular responses, Tut et al. (2021) showed that many participants had detectable responses and the responses were similar between younger and older participants, which is a great consideration in relation to T cells’ potential ability [137]. In addition, no significant difference was observed between BNT162b2 (Pfizer-BioNTech) or ChAdOx1 nCoV-19 (University of Oxford-AstraZeneca) vaccines.

Wang et al. (2021) reported a cohort of 63 individuals who had recovered from COVID-19 and were assessed at 1.3, 6.2 and 12 months after SARS-CoV infection; 43% of the cohort had received mRNA vaccines [140]. Wang and colleagues observed a notable evolution of neutralizing breadth after infection and a robust enhancement of serologic responses and B cell memory achieved with mRNA vaccination, which suggests that convalescent individuals who are vaccinated should have high levels of protection against VOCs without a need to modify existing vaccines. If a similar process occurs with naive individuals who receive vaccines, an additional dose should lead to protective immunity against circulating variants.

Likewise, Muena et al. (2021) studied 27 seropositive individuals who were immunized with the two main vaccines currently being used in Chile: CoronaVac (SinoVac) or BNT162b2 (Pfizer-BioNTech) vaccines [141]. Immunization of previously infected individuals with CoronaVac showed no significant differences in neutralizing antibody (NAb) titres after the first dose or when comparing the first and second doses. Significantly increased NAb titres were observed after both doses of CoronaVac, suggesting that infection induces a robust B-cell memory response. On the other hand, vaccination of seropositive individuals with mRNA and adenovirus-based vaccines induced high NAb titres after the first dose [87,98].

Therefore, vaccinated individuals who were infected with SARS-CoV-2 are less likely to transmit the virus to others than unvaccinated individuals, since they have a strong and rapid immune response against COVID-19. Besides, we can assume that the chance of transmitting the virus by vaccinated individuals who had the infection may decrease in relation to individuals who were only vaccinated.

## 5. Immune Correlates of Protection from SARS-CoV-2 Infection

One of the most frequently asked questions after recovery from COVID-19 or after completing the anti-COVID-19 vaccination schedule is about the degree of protection provided by contact with pathogen antigen. In fact, an effective immune response is important for preventing infection and/or disease. Knowledge of the levels of protection in a population may guide public health interventions, such as reopening decisions, maintenance or not of social distancing in a population, and the need for booster vaccination. In COVID-19, however, many aspects of immune correlates of protection are still unknown.

In brief, a correlate of protection is defined as an immune response statistically related to and/or responsible for protection. Once the immune system can be redundant, determining a correlate of protection is often complex, since more than one response may be a correlate, acting synergistically for protection [142]. Additionally, depending on the endpoint used, the correlate can refer to protection from infection, disease, severe disease or mortality [143].

Protection can be achieved through both natural infection and vaccines. In the case of SARS-CoV-2 infection, while natural infection can induce an immune response against the entire viral antigenic repertoire that directly stimulates the immune response, vaccines induce a systemic immune response that can target specific viral components, usually spike proteins, depending on the type of vaccine [144]. Although the nature of the stimulus may be different, studies suggest that in both cases, infection or vaccination, there is the development of an antibody response capable of generating protection [144,145,146].

To establish a correlate of protection, research in nonhuman primate models demonstrated that neutralizing antibodies promote protection against the SARS-CoV-2 challenge in a dose-dependent manner. Additionally, CD8+ T-cell depletion studies suggest that the cellular immune response participates in protection [147]. These data emphasize the importance of both humoral and cellular immunity for protection against SARS-CoV-2 and provide clues of possible correlates of protection from SARS-CoV-2 infection.

In addition, studies in cohorts with large numbers of individuals have had similar findings. A longitudinal study of a cohort of health workers recovered from COVID-19 showed that the presence of anti-SARS-CoV-2 antibodies was associated with a reduced risk of infection over 31 weeks, suggesting that previous infections resulting in specific antibodies can be associated with protection from reinfection [146]. Additionally, a recent publication has shown evidence for antibodies as protective correlates for COVID-19 vaccines. The authors evaluated seven vaccines currently in use worldwide (Pfizer, Moderna, Gamaleya, AstraZeneca, Sinovac, Novavax, and Johnson & Johnson) for efficacy and antibody induction. A robust correlation between antibody titres and efficacy was observed, with higher titres correlating with higher vaccine efficacy [78], corroborating antibodies as correlates of protection.

On the other hand, the durability of immunity to SARS-CoV-2 remains unclear since there is a decline in circulating antibody titres a few months after recovery or vaccination [148,149,150]. In turn, some authors have found sustained T-cell immunity despite a decline in the antibody response months after infection [151,152], suggesting that other immune components contribute to protective immunity.

The determination of serological antibody levels, which tests are accessible and practical, are the most commonly adopted correlates of protection for most infections. However, while serological assays detect circulating antibodies, which naturally decay in the absence of antigenic stimulation, cellular assays involve the restimulation of cells, enabling access to a cellular memory profile, even in the absence of antibodies. It is important to emphasize that the determination of the cellular response profile is more complex, involves the participation of a large variety of immune cells and cytokines, and requires cell culture assays involving antigenic stimulation. Among the huge repertoire involved, determining which cell subtypes and which cytokines are the best markers of immunological memory is still a challenge to be overcome. In this sense, although the assessment is more complex, the cellular response must be considered. Understanding these aspects is critical to know the duration of the immune response.

COVID-19 is a new disease, so many aspects remain to be determined. Thus, future studies should clarify, among many other aspects, the levels of circulating antibodies that offer effective protection from infection, how long immunity persists and protects against infection and the effect of the circulation of new variants.

## 6. Vaccination and Herd Immunity

Despite some undesirable side effects associated with COVID-19 vaccines [153] (most common ones described in Table 1), mass vaccination is likely the main strategy to guide us to the end of this worldwide unprecedented tragedy [154]. In this case, it is well understood that all advantages of SARS-CoV-2 vaccination overcome the risks of possible adverse events.

Many studies have discussed the efficiency and safety of COVID-19 vaccines, and we clearly noticed positive results in countries with advanced vaccination rates [155]. Confirmed cases, deaths and hospital admissions are sharply decreased in countries with a major population fully vaccinated [156]. Bauer et al. (2021) reinforced that the safest long-term strategy is keeping a low number of COVID-19 cases to preserve reduced mortality and morbidity. This strategy creates better conditions in the case of emerging SARS-CoV-2 variants that can escape the immune system and as governments reduce restrictive measures with progressing vaccinations [157].

Consequently, rising cases reinforce the importance of vaccination. In this way, an Italian study investigated SARS-CoV-2 resurgence in late 2020, in addition to the emergence of highly infectious variants. After using a Susceptible-Exposed-Infectious-Removed (SEIR) model, they emphasized the importance of preserving population-wide interventions, and considering the immunity decrease, seasonal vaccination would probably be necessary to control the pandemic [158].

Thus, in an attempt to ensure a collective goal, much has been discussed about herd immunity, which will occur when a significant portion of the population is immune to SARS-CoV-2, so that the spread of the virus is decreased, and more people are protected. Oh et al. (2021) detailed some challenges surrounding herd immunity, and they emphasized that a booster dose may be necessary once SARS-CoV-2 variants, which are able to evade immunity and have higher transmissibility, emerge. These authors also affirm that the COVID-19 overall response strategy will need to change according to the evaluation of population immunity. They assert that face mask-wearing and social distancing will still be required because while the COVID-19 pandemic will end, the virus will not disappear [159]. 

In addition, there is a consistent concern about protection among specific groups. Andryukov and Besednova (2021) reviewed strategies and platforms to improve the effectiveness of vaccination in older adults, as they are the most affected and vulnerable people because of immunosenescence and the consequent decrease in the effect of immunization [160]. Another public health concern is immunocompromised and immune-mediated inflammatory patients, who deserve special attention after vaccination, generally regarding whether patients on biologics will generate a sufficient immune response to the vaccine [161]. To address this issue, the UK’s Joint Committee on Vaccination and Immunisation (JCVI) has recommended that people with severely weakened immune systems should have a third vaccine dose [162]. Findings after a six-month follow-up in a cohort of HCW in Belgium also suggested the third dose scheme for individuals who were seronegative before vaccination [163]. 

Indeed, public health campaigns are crucial to improve COVID-19 vaccination rates and to reach individuals who are vaccine hesitant [164]. An insight is making vaccination easy and promoting autonomy by simplifying services and offering the public choices, so it may increase uptake in those who remain deliberative [165]. Having a skilled and knowledgeable health care team, as well as an open dialogue between patients and these professionals, is fundamental to the creation of a vaccine-positive culture. Then, we could easily identify barriers and determine solutions to improve vaccine uptake [166]. Undoubtedly, the influence of vaccine hesitancy is closely linked to skepticism and misinformation, and social media is currently a major contributor to this damage [167]. Additionally, Doustmohammadi and Cherry (2020) suggest that a long-term approach would be to introduce science and epidemiological education in grade school to college.

In summary, avoiding severe COVID-19 through vaccination outweighs the possible risks of vaccines. Regardless of vaccine type, misinformation in the form of fake news, and criticism of governments as neglectful and irresponsible in pandemic management, a vaccine-positive culture is key. It is the role of the scientific community to demystify and encourage those who remain hesitant to cooperate with the worldwide goal: to end the COVID-19 pandemic.

## 7. Final Considerations

Due to its leading role in infections, the immune system has been the focus of many studies in the context of COVID-19. From the dark, early days of the new virus and its effects on the human body, science unveiled in a worldwide effort many of the immunopathological and immunoprotecting aspects of the virus and improved disease treatment and the development of vaccines. Despite this progress, the durability of immunity to SARS-CoV-2 remains unknown and many aspects of COVID-19 require clarification. The safest long-term strategy is keeping low case numbers of COVID-19 to preserve reduced mortality and morbidity, and the best way to realize this goal is to support mass vaccination.

## Figures and Tables

**Table 1:** Characteristics of COVID-19 Vaccines Approved by the World Health Organization (WHO).

Platform.	Vaccine	Sponsor/Manufacturer	Doses	Adverse Reactions	Effect. (%)	Immune Response	Ref.
Inactivated virus	BBIBP-CorV	Sinopharm + China National Biotec Group Co. + Beijing Institute of Biological Products	2	Arthralgia, chills, fatigue, fever, headache, injection site pain, lymphadenopathy, myalgia, nausea/vomiting and swelling	79%	Humoral responses were induced on day 42 and two-dose immunization achieved higher neutralizing antibody titres than the single one	[70,83,84,85]
	CoronaVac	Sinovac Research and Development Co.	2	Injection site pain	50.7%, 83.7% and 100% against mild, moderate and severe infections, respectively	Right after the second-dose vaccine the seroconversion of neutralizing antibodies was seen for more than 90% of participants in a phase 1/2 clinical trial	[70,83,86]
mRNA- based	BNT162b2	Pfizer/BioNTech + Fosun Pharma	2	Chills, cough, diarrhea, fatigue, fever, headache, muscle and injection site pains, redness, shortness of breath, sore throat, vomiting and thrombotic events.	95%	Presented spike-specific IgG antibody production and ACE2 antibody binding inhibition responses; high levels of humoral and T-cell responses in an Asian population; elicits an adaptive humoral and poly-specific cellular immune response against epitopes that are conserved in a broad range of variants, at well-tolerated doses	[70,84,87,88,89,90]
	mRNA-1273	Moderna + National Institute of Allergic and Infectious Diseases (NIAID)	2	Arthralgia, chills, fatigue, fever, headache, injection site pain, lymphadenopathy, myalgia, nausea/vomiting, swelling and thrombotic events.	94%	Despite a slight expected decline in titres of binding and neutralizing antibodies, mRNA-1273 has the potential to provide durable humoral immunity, and also elicited primary CD4 type 1 helper T responses 43 days after the first vaccination	[70,84,90,91]
Viral Vector	AZD122 (ChAdOx1-S)	AstraZeneca + University of Oxford	2	Chills, diarrhea, fatigue, fever, headache, injection site pain, nausea, tenderness and thrombotic events.	70%	Induction of a Th1-biased response characterized by IFN-γ and TNF-α cytokine secretion by CD4+ T-cells and CD8+ T-cell were induced, both after a single dose in a phase 1/2 clinical trial; Increased anti-spike neutralizing antibody titres, Fc-mediated functional antibody responses, antibody-dependent neutrophil/monocyte phagocytosis, complement activation and natural killer cell activation with two-dose vaccine regime.	[83,92,93,94]
	Ad26.COV2.S	Janssen Pharmaceutical by Johnson & Johnson	1	Fatigue, fever, headache, injection site pain and myalgia	66%	Neutralizing-antibody titres against wild-type virus were detected in up to 90% of the participants on day 29. Titres remained stable until at least day 71. Both cases occurred after the first vaccine dose in a Phase 1–2a trial. In addition, on day 15, CD4+ T-cell responses were detected up to 60% of the participants with a clear skewing toward type 1 helper T-cells. CD8+ T-cell responses were robust overall.	[74,83,95]
	Convidicea	CanSino Biological Inc./ Beijing Institute of Biotechnology	1	Fatigue, fever, headache, injection site pain, muscle pain and myalgia	65.7%	Humoral responses peaked at day 28 and rapid specific T-cell responses were noted from day 14 post-vaccination.	[84,96]

Effect.: effectiveness; Ref.: reference.

## Data Availability

Not applicable.
